# Supplementary material for: The Chemical Deformation of a Thermally Cured Polyimide Film Surface into Neutral 1,2,4,5-Benzentetracarbonyliron and 4,4′-Oxydianiline to Remarkably Enhance the Chemical–Mechanical Planarization Polishing Rate
Source: Nanomaterials (Basel). 2025 Mar 10;15(6):425. doi: 10.3390/nano15060425 (PMC11944291; doi:10.3390/nano15060425)
Supplement: Supplementary file 1 [file nanomaterials-15-00425-s001.zip › nanomaterials-3450031-supplementary.pdf]

Supplementary Materials

The Chemical Deformation of a Thermally Cured Polyimide Film Surface into Neutral 1,2,4,5-benzentetracarbonyliron and 4,4′-oxydianiline to Remarkably Enhance the Chemical–Mechanical Planarization Polishing Rate

Man-Hyup Han [1,†], Hyun-Sung Koh [2,†], Il-Haeng Heo [2,†], Myung-Hoe Kim [2], Pil-Su Kim [1], Min-Uk Jeon [2], Min-Ji Kim [2], Woo-Hyun Jin [2], Kyoo-Chul Cho [2], Jinsub Park [2] and Jea-Gun Park [1,2,*]

[1] Department of Nanoscale Semiconductor Engineering, Hanyang University, Seoul 04763, Republic of Korea; aksguq06@hanyang.ac.kr (M.-H.H.); rhgustjd09@gmail.com (P.-S.K.)

[2] Department of Electronic Engineering, Hanyang University, Seoul 04763, Republic of Korea; psk6208@naver.com (H.-S.K.); heo5170@naver.com (I.-H.H.); ck05102@naver.com (M.-H.K.); mu-jeon1214@gmail.com (M.-U.J.); a2024157411@hanyang.ac.kr (M.-J.K.); jinwh7@naver.com (W.-H.J.); kccho12@naver.com (K.-C.C.); jinsubpark@hanyang.ac.kr (J.P.);

* Correspondence: parkjgl@hanyang.ac.kr

† These authors contributed equally to this work.

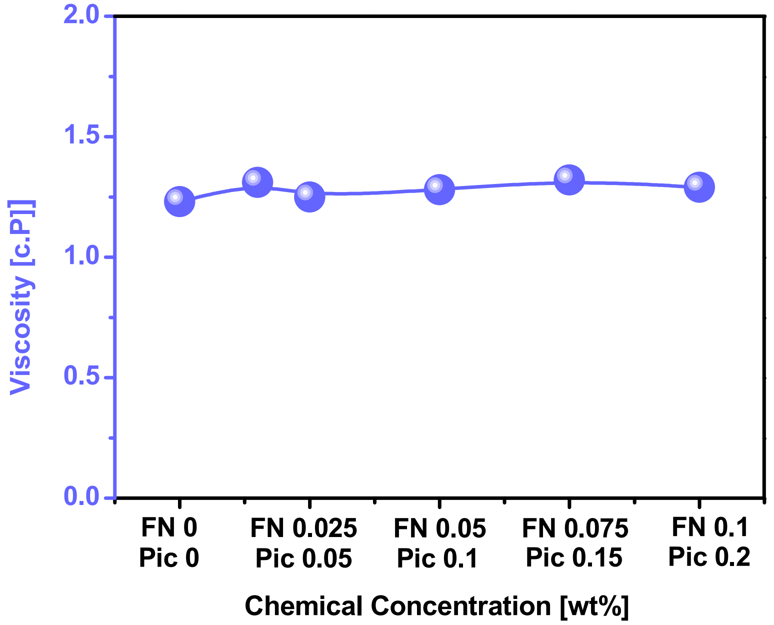

Figure S1. Dependency of the slurry viscosity on the ferric catalyst concentration for the PI film CMP slurry.

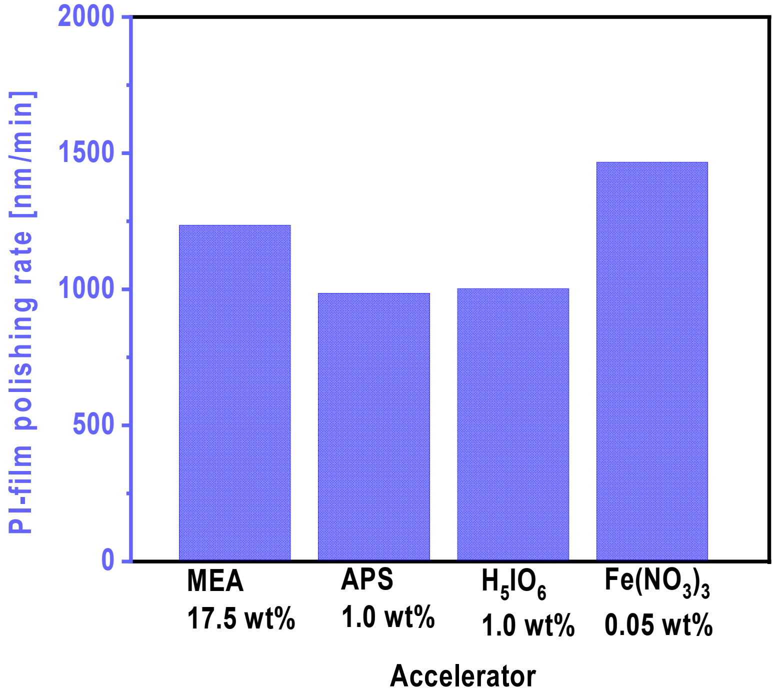

Figure S2. Dependency of PI film polishing rates on the accelerator types in the slurries including ethanol amine, ammonium persulfate, periodic acid, and $Fe(NO_3)_3$.

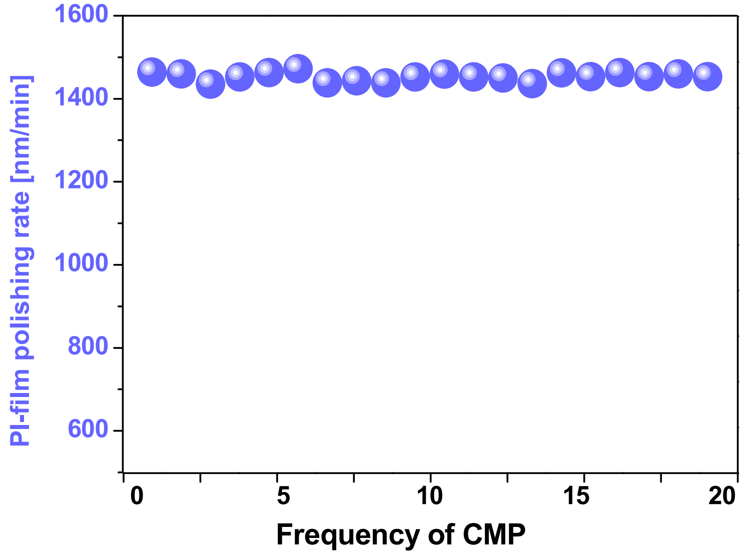

Figure S3. Dependency of the PI film polishing rates on the frequency of CMP.
